# The Influence of Recycling on the Properties of Interface between Ceramic and Dental Alloys

**DOI:** 10.1155/2020/3529781

**Published:** 2020-04-08

**Authors:** Yinghui Wang, Honglan Huang, Honglei Lin, Lei Jiang, Yu Pan, Xiurong Li, Hui Cheng

**Affiliations:** ^1^Fujian Key Laboratory of Oral Diseases & Fujian Provincial Engineering Research Center of Oral Biomaterial & Stomatological Key lab of Fujian College and University, School and Hospital of Stomatology, Fujian Medical University, Fuzhou, Fujian, China; ^2^Institute of Stomatology & Research Center of Dental Esthetics and Biomechanics, School and Hospital of Stomatology, Fujian Medical University, Fuzhou, Fujian, China; ^3^Stomatological Hospital of Xiamen Medical College, Xiamen, Fujian, China; ^4^Department of Prosthetic Technology, School and Hospital of Stomatology, Fujian Medical University, Fuzhou, Fujian, China

## Abstract

**Purpose:**

The purpose of this study was to evaluate the effect of recycling on the properties of interface between 2 dental alloys and their corresponding porcelains.

**Materials and Methods:**

Noble alloy (Pd-Cu-Ga) and high-noble alloy (Au-Pt) were used in this study. Metal matrices (cylinders *Φ*4 mm × 4 mm with pedestal *Φ*5 mm × 1 mm) were prepared by arc melting in argon after recasting 1-3 times. Corresponding porcelain with overall dimensions of *Φ*4 mm × 2 mm was veneered on each metal cylinder. There were 22 specimens in each alloy group. Specifically, two specimens of each group were chosen randomly for interfacial morphology and diffusion analyses by scanning electron microscopy (SEM) equipped with energy-dispersive X-ray spectroscopy (EDS). The remaining 20 specimens were divided into 2 groups with or without thermal cycling. The bond strength was evaluated by shear test, and the data were analyzed by two-way analysis of variance (ANOVA). The failure mode of shear test specimen was observed with a stereoscopic microscopy and subjected to the exact probability test (*α* = 0.05).

**Results:**

According to the results from SEM, no obvious difference was observed in the interfacial morphology of both Pd-Cu-Ga and Au-Pt alloys among different recasting specimens. EDS analysis revealed that no significant difference was found in the width of elemental diffusion among 2 test alloys after recycling 1-3 times. Notably, in Pd-Cu-Ga alloy groups, the peak of Ga in thrice recasting was lower than those in first and second recastings. And there was no significant difference (*P* > 0.05) in the metal-ceramic shear bond strength of Pd-Cu-Ga and Au-Pt alloys after recycling 1-3 times, with or without thermal cycling. The results of failure modes observed on specimens were not affected by the recycling and thermal cycling in the 2 tested alloys.

**Conclusions:**

Within the limitations of this study, the Pd-Cu-Ga and Au-Pt alloys can be recycled 2 times without significant changes on the properties of metal-ceramic interface, with or without thermal cycling.

## 1. Introduction

As all-ceramic restorations, CAD/CAM and 3-dimensional printing technology are now available in dental clinics; conventional casting metal-ceramic restorations are not the only choice for dentists and patients. However, compared to metal-ceramic restorations, the long-term outcomes and complications of these new restorations are still not well understood. For example, the incidence rate of catastrophic framework fracture and veneer chipping in all-ceramic restorations is higher than similar metal ceramic restorations [1, 2], and there is still no CAD/CAM and 3-dimensional printing technology with noble and high-noble alloys. Therefore, conventional casting of noble and high-noble alloys metal-ceramic restorations will still likely remain as one of the proven restorative options for patients in the near future.

However, the conventional lost-wax metal casting process of metal-ceramic restorations usually produces a large amount of dental alloy waste, such as the sprues and cast investments, thus increasing medical costs, wasting metal resource, and polluting the environment as well. So nowadays, noble and high-noble alloys are commonly recycled in dental laboratories.

A number of authors investigated the feasibility of recycling dental alloys, but these studies varied in research methodology and designs. There was no agreeable test and evaluation protocol for recycling yet. [3] Our recent study has established a series of mechanical and chemical treatments for previously melted buttons or sprues before recycling, and the results showed that the contamination of previously cast ceramic alloys could be effectively removed. [4] After recasting thrice without adding any new alloys by electric arc melting under an argon gas-protective vacuum environment, we found that there was no significant decline of tensile strength, 0.2% yield strength, flexural strength, flexural modulus, and Vickers hardness in Co-Cr and Au-Pt alloys but decrease of tensile properties in cpTi [5–7].

In addition to the mechanical properties, the metal-ceramic compatibility is also of great importance to the clinical success. Hence, the purpose of this study was to evaluate the effect of recasting on the properties of interface between 2 dental alloys (noble and high-noble) and their corresponding porcelains after recasting 1-3 times.

## 2. Materials and Methods

The present study tested noble alloy (Pd-Cu-Ga) and high-noble alloy (Au-Pt) after recasting 1-3 times, with or without thermal cycling. Manufacturers and composition of the ceramic alloys used are shown in Table 1.

### 2.1. Specimens Preparation

132 wax specimens (cylinder *Φ*4 mm × 4 mm with a pedestal *Φ*5 mm × 1 mm) were randomly and equally divided into 6 groups (*n* = 22) as shown in Table 2. The wax specimens were sprued and invested with a phosphate-based investment (Vesto-Fix, DFS, Germany) according to the manufacturer's instructions. Then 2 ceramic alloys were melted in individual crucibles under an argon gas-protective vacuum environment. After the castings had been cooled at room temperature, they were airborne-particle abraded with 120 *μ*m Al_2_O_3_ and cleaned in distilled water to remove residual investment on the surface. Next, alloy specimens were separated from the castings. The buttons and sprues from the first and second castings were prepared according to the treatment protocols that we have previously tested and published as the following [4]: (1) Pd-Cu-Ga ceramic alloys: glass bead airborne-particle abrasion and 30 mins immersion in 40% HF solution; (2) Au-Pt ceramic alloys: glass bead airborne-particle abrasion. No new alloys were added for the second or third castings for all groups.

**Figure 1:**
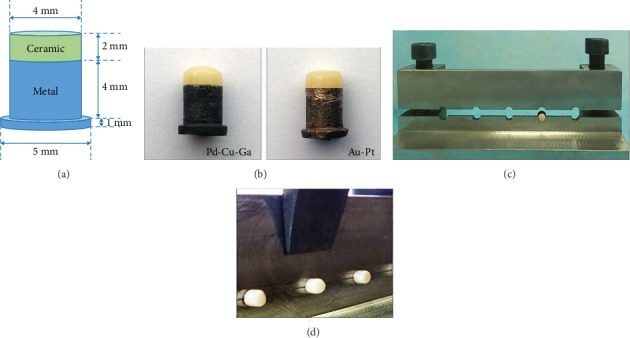
(a) Specimens of 2 tested alloys. (b) Dimensions of the ceramic-alloy specimens. (c) The holder of specimens for shear bond test. (d) The loading device of shear bond test.

The surfaces of specimens were polished with silicon carbide abrasive paper from 240 grit to 600 grit. Then the surface of each alloy was abraded by airborne particles as the following previously established protocols [4]: (1) Pd-Cu-Ga ceramic alloys: 120 *μ*m Al_2_O_3_ at an angle of 45° for 15 s from a distance of approximately 2 cm, under 0.2 MPa; (2) Au-Pt ceramic alloys: 180 *μ*m glass bead at an angle of 45° for 15 s from a distance of approximately 2 cm, under 0.2 MPa, and then ultrasonic cleaned in isopropyl alcohol and distilled water for 5 mins and drying at room temperature. Notably, the surface of Pd-Cu-Ga specimens were oxidized before airborne-particle abrasion. All the specimens were veneered using a costumed mold, including two layers of opaque and body porcelain, respectively, following with glazing as the last step (Table 3 and Figures 1(a) and 1(b)). The porcelain powder was VMK95 (VMK 95, Vita, Germany).

More specifically, 2 specimens of each tested alloy were chosen randomly for SEM observation of interfacial morphology and diffusion analysis. The remaining 20 specimens were divided into 2 groups: one with and the other without thermal cycling. The groups without thermal cycling were stored in distilled water for 24 h at 37°C, while the groups with thermal cycling were subjected to 3,000 thermal cycles (between 5 and 55°C, dwell time: 30 s, transfer time: 10 s).

### 2.2. Interfacial Morphology and Diffusion Analysis

According to the method of metallographic microscopic structure examination (GB-T13298-2015) [8], two specimens chosen randomly from each group were embedded in autopolymerizing acrylic resin, and then the lateral surface of the specimens was abraded with silicon carbide abrasive paper from 240 grit to 1,500 grit in order to expose the metal-ceramic interface. They were subsequently polished with diamond paste in the buffing machine with water coolant.

The interfaces of Pd-Cu-Ga specimens were gold-sprayed within an ion sputtering equipment, and then interface diffusion and morphology were assessed by SEM (SU-70, HITACHI, Japan) equipped with EDS analysis (Oxford, X-Max, Great Britain). Considering the influence of gold ions on interface diffusion analysis in Au-Pt specimens, interface diffusion analysis of them was conducted before gold spraying.

### 2.3. Shear Bond Strength Test

Specimens were subjected to the shear test at room temperature in a universal material testing machine (Model 1342, INSTRON, UK). The load was applied to the metal-ceramic interface at a constant speed of 0.5 mm/min, and the load on the test specimen was recorded at failure. (Figures 1(c) and 1(d)).

Shear bond strength was analyzed by two-way analysis of variance (ANOVA) and followed with Tukey honest significant difference (HSD) test. The data were analyzed by the Statistical Package Social Sciences (version 21) software (SPSS, Chicago, IL, USA), and *P* values less than 0.05 were considered statistically significant.

### 2.4. Failure Type Observation

Failure type ((1) cohesive failure, (2) adhesive failure, (3) mixed failure) of each specimen was determined by the stereoscopic microscopy (ZSA302, COIC, China).

Exact probability test was used to analyze whether recasting has an impact on the metal-ceramic failure type (*α* = 0.05).

## 3. Results

### 3.1. Interfacial Morphology and Diffusion Analysis

**Figure 2:**
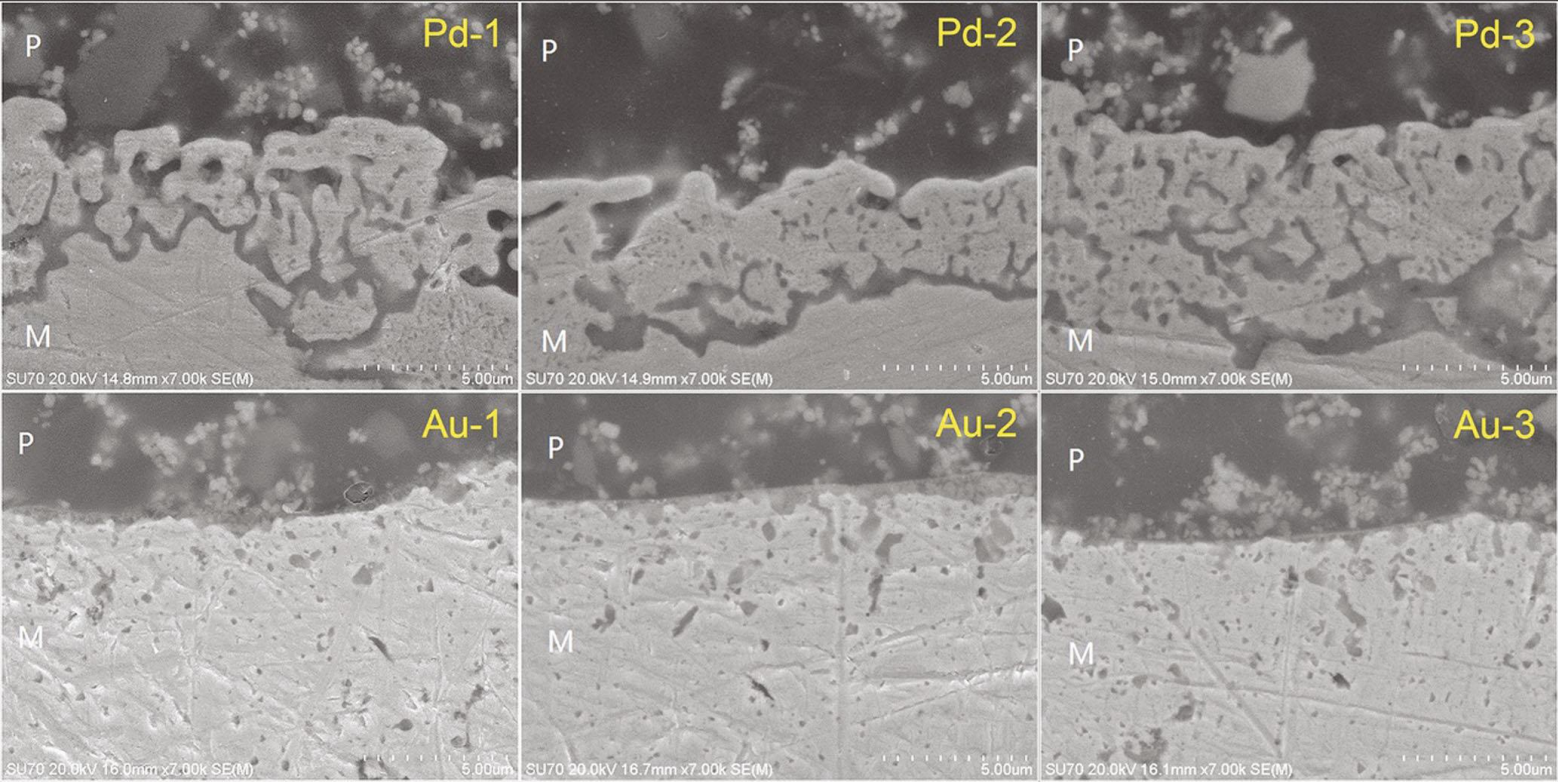
Morphology of metal-ceramic interface of Pd-Cu-Ga and Au-Pt (×7,000) alloys after recasting 1-3 times under SEM.

As shown in Figure 2, three different regions of specimens were measured, metal matrix (M), porcelain layer (P), and interfacial layer, according to the cross-sectional morphology under SEM. The results indicated that the metal-ceramic interfaces combined closely in most tested alloys without pores and cracks, indicating good wettability and an appropriate adhesion. Generally, no obvious difference was observed in the interfacial morphology of all tested alloys among different recasting times.

**Figure 3:**
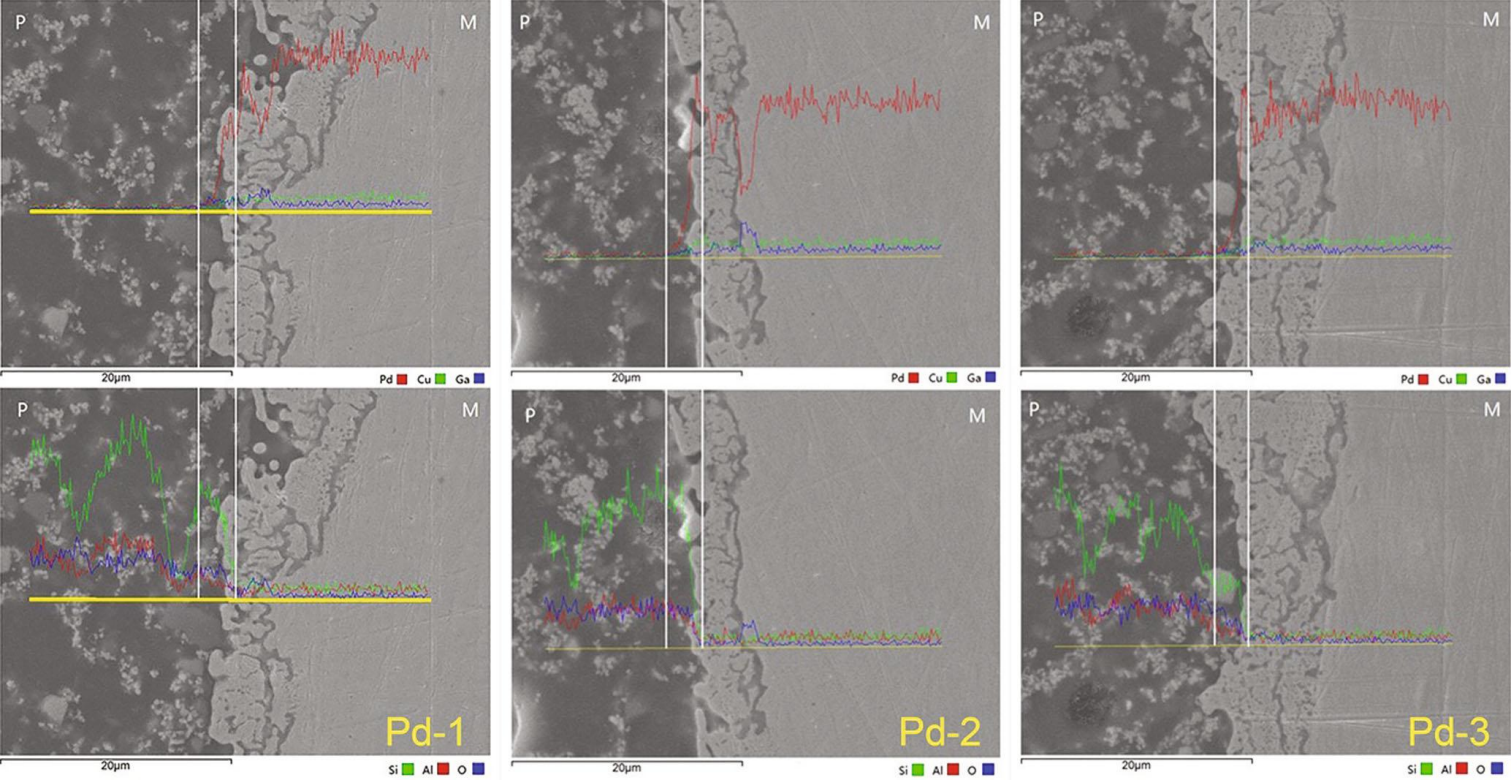
Metal-ceramic interfacial diffusion analysis of Pd-Cu-Ga alloy after recasting 1-3 times by SEM equipped with an EDS (×3,000).

**Figure 4:**
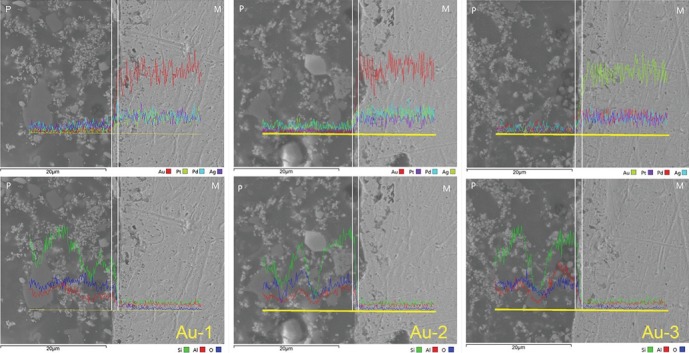
Metal-ceramic interfacial diffusion analysis of Au-Pt alloy after recasting 1-3 times by SEM equipped with an EDS (×3,000).

EDS analysis at the interface of all tested alloys after recasting 1-3 times revealed elemental diffusion across the metal-ceramic interface (Figures 3 and 4). In general, no significant difference was found in the width of elemental diffusion on 2 test alloys after recasting 1-3 times. Notably, in Pd-Cu-Ga alloy, the peak of Ga in thrice recasting was lower than those in first and second recastings.

### 3.2. Shear Bond Strength

**Figure 5:**
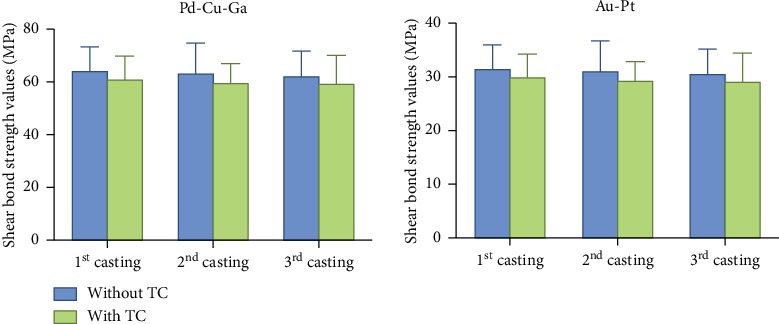
Shear bond strength of 2 tested alloys after recasting 1-3 times with and without thermal cycling.

As shown in Figure 5, there was no significant interaction between recasting and thermal cycling (*F*_Pd−Cu−Ga_ = 0.007, *P*_Pd−Cu−Ga_ = 0.993; *F*_Au−Pt_ = 0.074, *P*_Au−Pt_ = 0.929), and no significant difference in the metal-ceramic shear bond strength of Pd-Cu-Ga and Au-Pt alloys after recasting 1-3 times, whether subjected to the thermal cycling or not (Recasting: *F*_Pd−Cu−Ga_ = 0.177, *P*_Pd−Cu−Ga_ = 0.838; *F*_Au−Pt_ = 0.403, *P*_Au−Pt_ = 0.912; Thermal cycling: *F*_Pd−Cu−Ga_ = 1.598, *P*_Pd−Cu−Ga_ = 0.212; *F*_Au−Pt_ = 0.538, *P*_Au−Pt_ = 0.466).

### 3.3. Failure Type Observation

**Figure 6:**
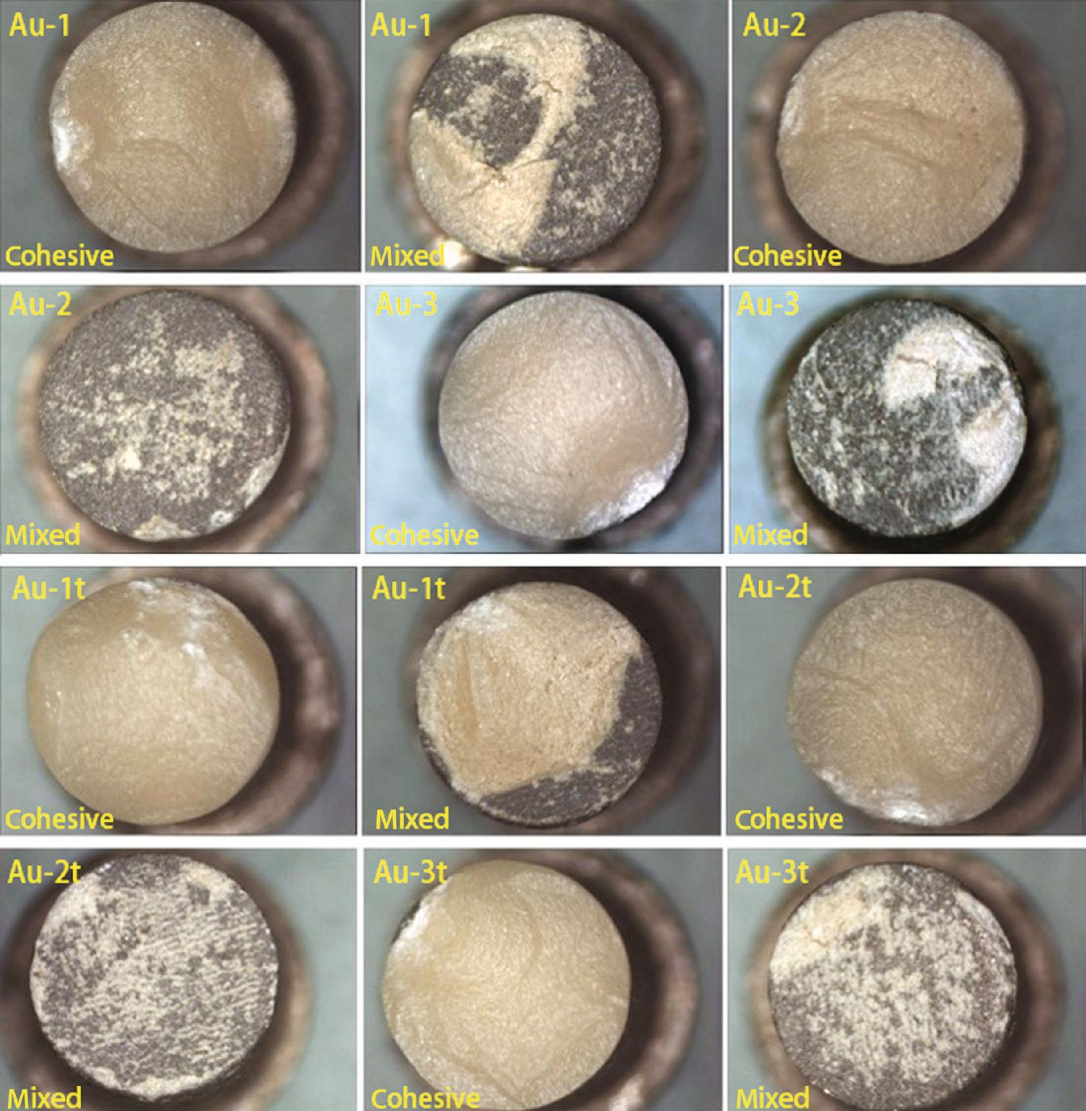
Failure type of Au-Pt alloy after recasting 1-3 times with or without thermal cycling.

**Figure 7:**
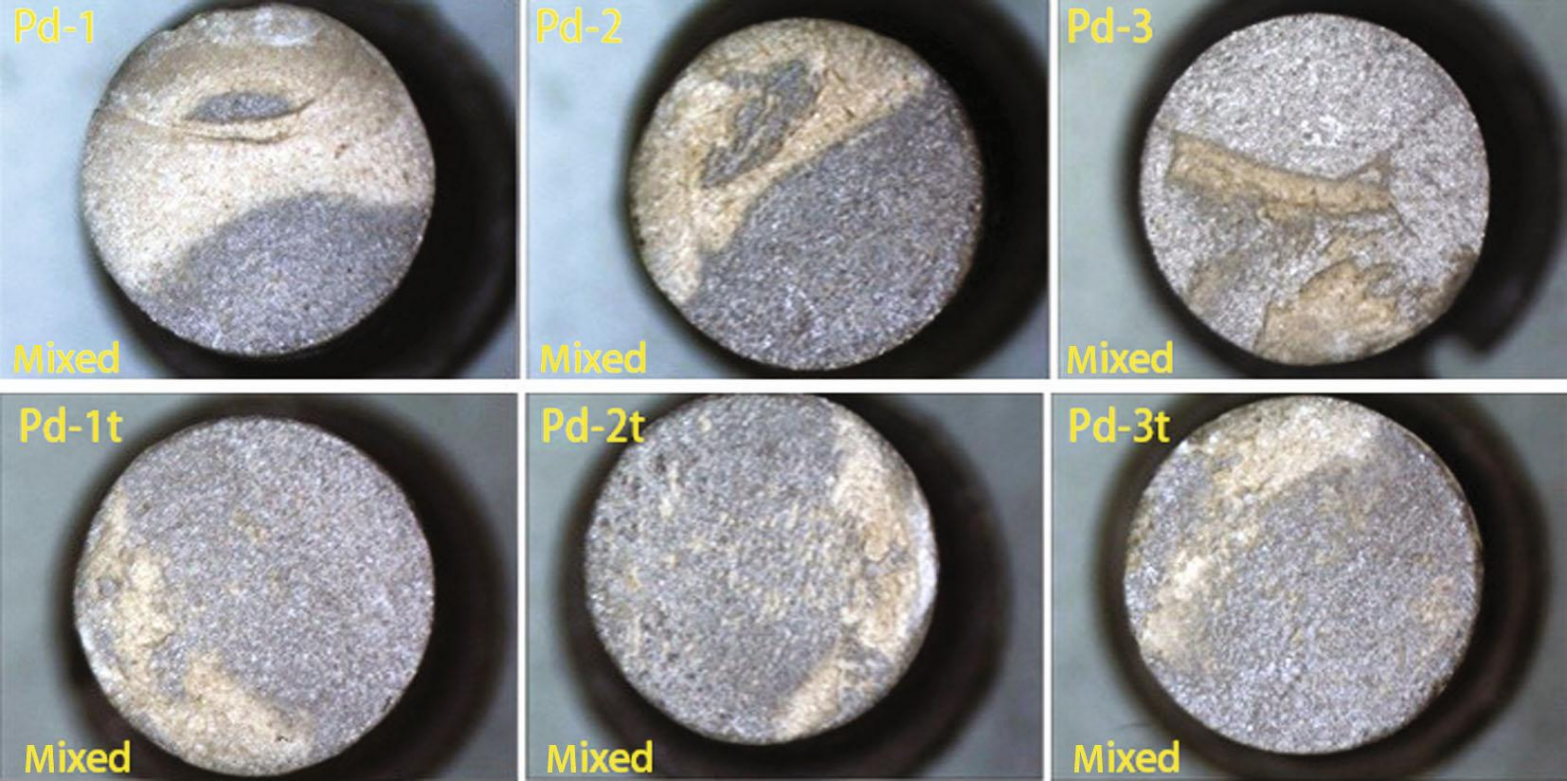
Failure type of Pd-Cu-Ga alloy after recasting 1-3 times with or without thermal cycling.

The fracture types were classified according to the presence or not of ceramic remnants on the metal substrate after shear tests. It was described as follows: (1) adhesive, if no remnants of ceramic were found on the metal surface; (2) cohesive, if fracture occurred within the ceramic side; and (3) mixed, if remnants of ceramic were found in the metal surface. In this study, there was a few cohesive fracture modes in the Au-Pt group (Figure 6), and only mixed mode was found in Pd-Cu-Ga group. (Figure 7).

**Figure 8:**
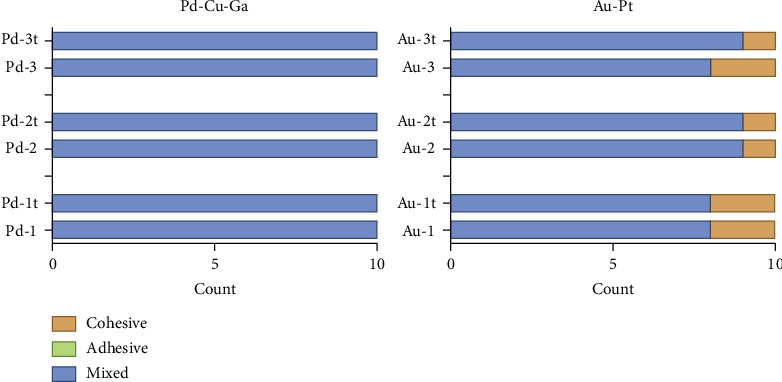
Failure type of 2 tested alloys after recasting 1-3 times with or without thermal cycling.

According to the exact probability analysis, there was no significant difference (*P* > 0.05) in the metal-ceramic restorations failure type of all tested alloys after recasting 1-3 times, whether subjected to the thermal cycling or not. (Figure 8).

## 4. Discussion

In this study, a favorable metal-ceramic combination of Pd-Cu-Ga and Au-Pt alloy-ceramic restorations was observed, and the width of the oxide layer analyzed by elemental diffusion did not change significantly on 2 ceramic alloys after recasting 1-3 times. These indicated that recasting 2 times had no obvious negative effects on the oxidation zone of metal-ceramic. It is known that the metal-ceramic bonding is fundamentally based on micromechanical retention, compressive adaptation, chemical union, and Van der Waals forces [9]. The oxide zone is important for forming chemical combination between metal and porcelain, and its thickness directly affects the metal porcelain bond strength. If the oxide layer is thick or loose, its thermal expansion coefficient will mismatch with the ones of metal and porcelain, resulting in residual stress in the interface and decreased bond strength. On the other hand, if the oxide layer is too thin, it will be completely soluble in porcelain in the sintering process, making direct contact of porcelain with the metal base by a van der Waals force rather than the chemical bonding, and this also reduces the metal-ceramic bond strength.

The results of shear bond strength and failure type further verified the results of interfacial morphology and diffusion analysis. No significant difference was found in the metal-ceramic bond strength of Pd-Cu-Ga and Au-Pt alloys after recasting 1-3 times with or without thermal cycling. Consistently, the results of failure type observed on specimens were not affected by recasting and thermal cycling in the 2 ceramic alloys. Although 3-point bending test was recommended by ISO 9693 for evaluating the metal-ceramic bond strength, some authors considered the shear test to be a more adequate and reliable method because there were less variables and residual stress at the metal-ceramic interface. [10–13] In addition, the results of the shear test are not influenced by Young's modulus of the alloy. [14] Moreover, Ucar et al. [15] found that the 3-point bending test and shear test shared a similar result in the metal-ceramic bond strength. Therefore, we chose to conduct shear test to determine the difference of bond strength among the alloys after recasting 1-3 times.

To explore the causes of these study results, there are two possible reasons. One is melting method in different studies. Ceramic alloys will likely change their properties after recasting resulting from evaporation, oxidation, or contamination in the process of melting. In this study, the melting method was arc melting with argon. It was a more favorable condition for recasting alloys because argon is hard to dissolve in molten liquid alloy or to react with the component of alloys. This will effectively protect the components of alloys from oxidation. In the study of Peraire et al. [16], after recasting high noble and noble alloys 7 times in a vacuum casting condition, the components of noble alloys and the major constituents of high noble ceramic alloy remained stable. The other possible reason is the treatment of previously melted ceramic alloys before recasting. Most of the contamination in previously cast alloys come from investment materials, oxides (such as Al_2_O_3_, Fe_2_O_3_ and SiO_2_), reaction layers, and airborne-particle abrasion with Al_2_O_3_. In this study, previously cast alloys were treated before recasting following the established protocols from our group, which successfully removed the contamination from recasting process and airborne-particle abrasion. [4] Compared to the new alloys after polishing, the impurity element was not detected in previously cast alloys after being treated. Hence, the combined effects of arc melting with argon and the treatment of ceramic alloys before recasting might be the underlying reasons for the results of our study.

The metal-ceramic bond strength would be strong or weak if the fracture type was cohesive or adhesive, respectively. Compared with the fracture mode with the bond strength, it seemed that there was no direct correlation between the type of failure and the bond strength. When it comes to the thickness of metal-ceramic reaction zone, we could find Pd-Cu-Ga alloy was higher than the Au-Pt alloy. Therefore, we can speculate that the thickness of metal-ceramic interfacial region might be the related factor of the failure mode, but it still needs to be further researched.

However, even without significant differences, there was still decline in the bond strength of each alloy after recasting 1-3 times based on the data in this study. The inevitable element loss in the melting process might be responsible for the slight decline of metal-ceramic bond strength. For example, in the Pd-Cu-Ga group, the peak of Ga in third recasting, which is the foundation of the metal-ceramic combination by bonding with Cu [17, 18], was lower than that in first and second recastings. It also has been reported that after recasting 7 times in a vacuum-conditions, Zn and Sn in high noble alloy declined significantly by 50% and 100%, respectively. Thus, it implied that the quality of metal porcelain bonding might be reduced if the number of recasting exceeds two or three.

Thermal cycling, which is commonly utilized to simulate oral long-term condition in vitro, can trigger repetitive stress on metal-ceramic interface, thus weakening the bond strength [19–21]. Moreover, discrepancy between the thermal expansion coefficients of the two combined materials during the process of thermal cycling can also affect adhesive strength. Hence, thermal cycling is considered a way of aging the ceramic-metal interface [22, 23]. In addition, 3,000 thermal cycles were reported to be equivalent to 2.5 years of clinical application [24]. Based on these conditions, that means that metal-ceramic compatibility of 2 tested alloys was not significantly affected by recasting 1-3 times in short-medium term aging. However, long-term aging effect should be further studied.

## 5. Conclusions

Within the limitations of this study, Pd-Cu-Ga and Au-Pt ceramic alloys can be recycled 2 times without significant decrease on metal-ceramic interface properties, with or without thermal cycling. But giving thought to the labor and material costs of the treatment protocols with the alloys previously cast, it is more recommended that hulk alloys recycling is conducted by manufacturers.

## Figures and Tables

**Table 1:** Compositions of 4 ceramic alloys as provided by manufacturer (wt%).

Alloy types	Manufacturer	Composition (wt%)
Pd-Cu-Ga	Albabond E, HeraeusKulzer, Wertheim, Germany	Pd: 78.0, Cu: 10.8, Ga: 7.5, Au: 1.6, In: 1.3, Ru: 0.4, Zn: 0.2, Sn: 0.2
Au-Pt	Alfa Ceramic 90, Alldental, Stockholm, Sweden	Au: 89.5, Pt: 5.8, Pd: 1.6, Ag: 1.2, Ir: 0.6, Sn: 0.3, In: 0.8, Fe: 0.2

**Table 2:** Test specimen groups.

Alloy type	Thermal cycling	Cast once	Cast twice	Cast thrice
Pd-Cu-Ga	Yes	Pd-1t	Pd-2t	Pd-3t
	No	Pd-1	Pd-2	Pd-3

Au-Pt	Yes	Au-1t	Au-2t	Au-3t
	No	Au-1	Au-2	Au-3

**Table 3:** Firing procedures of the dental ceramics.

Ceramics	ST (°C)	DT (min)	TRI (°C/min)	FT (°C)	HT (min)
VMK95					
Oxidation	500	6	55	930	1
First opaque layer	500	2	80	950	1
Second opaque layer	500	6	55	930	1
First dentin layer	500	6	55	930	1
Second dentin layer	500	6	55	920	1
Glaze firing	500	2	55	915	1
Duceratin					
Bonder	600	6	100	750	1
First opaque layer	450	10	100	760	0.5
Second opaque layer	450	10	100	760	0.5
First dentin layer	450	9	55	760	1
Second dentin layer	450	8	55	750	1
Glaze firing	450	6	55	730	1

ST: starting temperature; DT: drying time; TRI: temperature rate of increase; FT: final temperature; HT: holding time.

## Data Availability

The data used to support the findings of this study are available from the corresponding author upon request.

## Additional Points

*Clinical significance*. After special treatment before recasting and under arc melting with argon, Pd-Cu-Ga and Au-Pt alloys can be recycled 2 times without significant changes in the metal-ceramic interface.
